# Prenatal diagnosis of Holt-Oram syndrome

**DOI:** 10.1515/crpm-2021-0058

**Published:** 2022-06-16

**Authors:** Virginia Foreste, Carla Riccardi, Brunella Zizolfi, Alessandra Gallo, Attilio Di Spiezio Sardo

**Affiliations:** Department of Neuroscience Reproductive Sciences and Dentistry School of Medicine, School of Medicine, University of Naples Federico II Naples, Naples, Italy; Department of Public Health, School of Medicine, University of Naples Federico II Naples, Naples, Italy

**Keywords:** congenital heart malformation, MRI, prenatal diagnosis, ultrasound

## Abstract

**Objectives:**

To detect common congenital disorders in Holt-Oram syndrome.

**Case presentation:**

We present a case of a 32 years old primigravida pregnant woman affected by Holt-Oram syndrome referred to our institution for second trimester routine anatomy scan. The ultrasound reported a bilateral aplasia radii, slightly curved ulna and bilateral twisted hand with four digital rays. A significant enlargement of the right atrium without tricuspid regurgitation was also detected. The patient refused the amniocentesis and the postnatal evaluation confirmed the diagnosis of Holt-Oram syndrome.

**Conclusions:**

Holt-Oram syndrome is an autosomal dominant genetic condition. It is characterized by abnormalities in the bones of the upper limb and congenital heart malformation. The mutation can be inherited, but most cases result from a new mutation in patients without family history of the disorder.

## Introduction

Holt-Oram syndrome is an autosomal dominant genetic condition. It is characterized by abnormalities in the bones of the upper limb and congenital heart malformation [1]. The only gene known to be associated with Holt-Oram syndrome is the *TBX5* gene. A *TBX5* gene mutation has been identified in approximately 74% of individuals affected with Holt-Oram syndrome [2]. Currently, there are more than 70 known mutations in the *TBX5* gene that cause Holt-Oram syndrome [3]. The mutation can be inherited, but most cases result from a new mutation in patients without family history of the disorder.

Here we present a case of prenatal diagnosis of Holt-Oram syndrome in a pregnant woman affected by the disease.

## Case presentation

**Figure 1:**
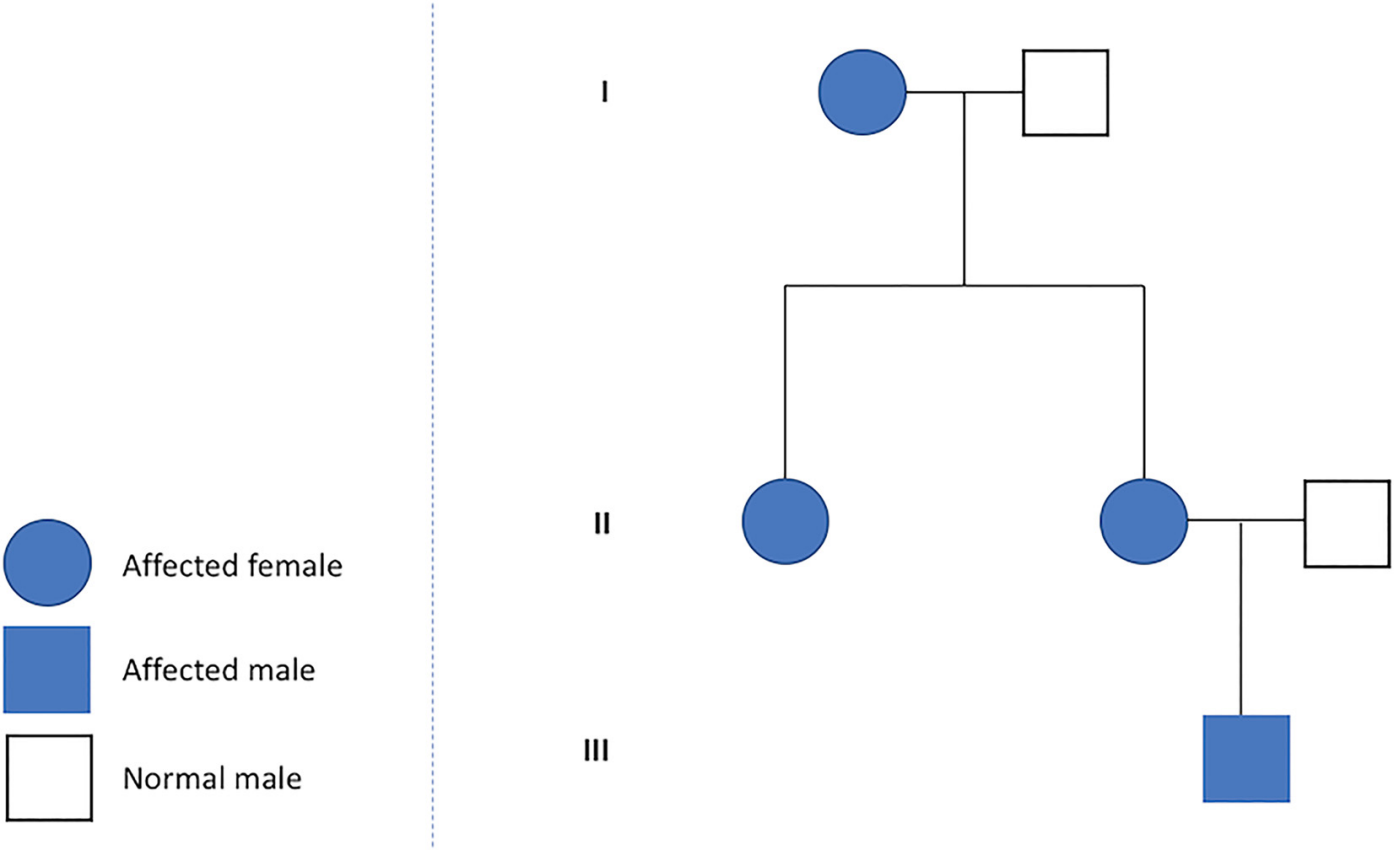
Family tree showing the autosomal dominant pattern of inheritance.

**Figure 2:**
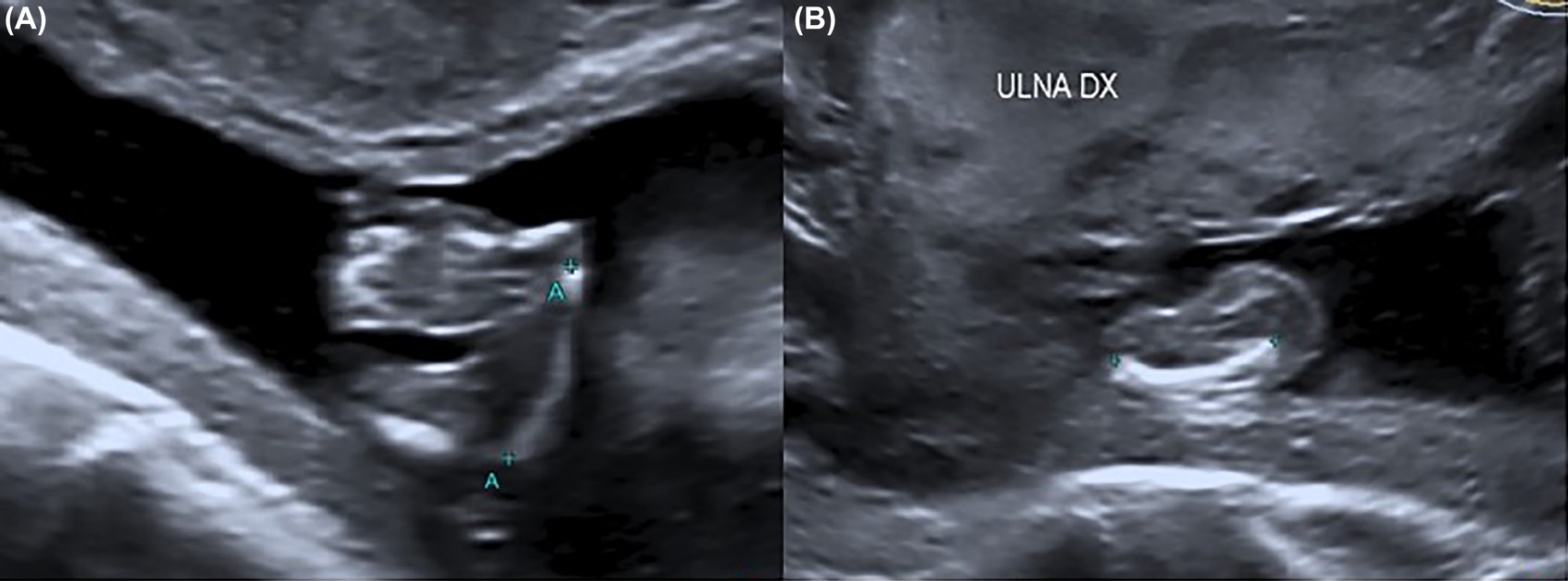
Ultrasonographic images of fetal upper limb defects at 18 weeks of gestation. (A) Twisted fetal hand. (B) Curved ulna and radio aplasia.

**Figure 3:**
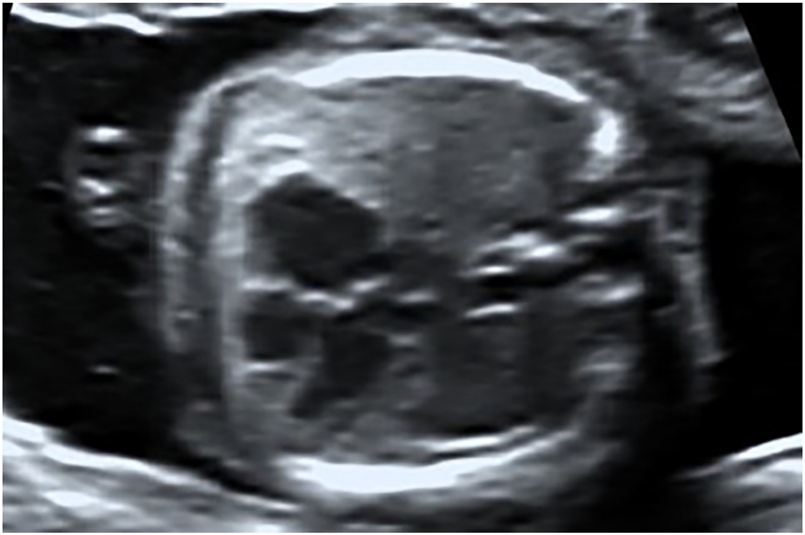
Enlargement of the right atrium.

**Figure 4:**
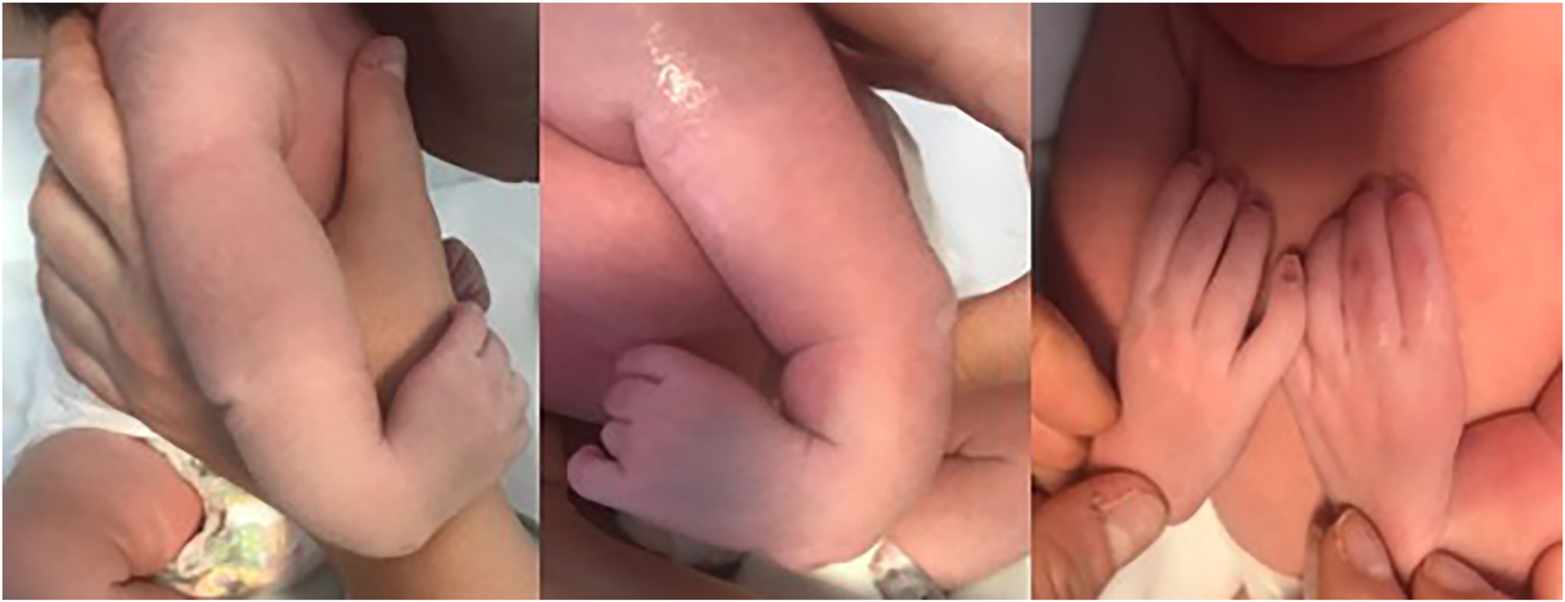
Upper limb defects at time of birth.

A 32 year old primigravida pregnant woman affected by Holt-Oram syndrome was referred to our institution for second trimester routine anatomy scan. She had a positive family history of Holt-Oram syndrome involving her mother and her sister (Figure 1). In all family members affected, the only clinical evidence of the syndrome regards the upper limb defects, with a carpal bone malformation and agenesis of the thumb. The ultrasound reported a bilateral aplasia radii, slightly curved ulna and bilateral twisted hand with four digital rays (Figure 2). A significant enlargement of the right atrium without tricuspid regurgitation was also detected (Figure 3). The patient refused the amniocentesis and decided to carry on with the pregnancy. At the time of follow-up ultrasound at 30 weeks, the right atrial enlargement was confirmed without tricuspid insufficiency or other cardiac malformations. The patient underwent emergent cesarean delivery at 40 weeks of gestation because of fetal bradycardia during labor. A male infant was delivered with a birth weight of 3,110 g and APGAR score of 9 and 9 at 1′ and 5′ min. Postnatal evaluation confirmed the severe radii hypoplasia, slightly curved ulna and agenesis of the first ray (Figure 4). Neonatal echocardiography ducted the right atrial enlargement noticing also an atrial and two little ventricular septal defects not detected prenatally. The genetic analysis was also performed, and *TBX5* mutation was detected.

## Discussion

Holt-Oram syndrome is a heart-hand syndrome characterized by abnormalities of the upper limbs and shoulder girdle associated with a congenital heart lesion [4]. It is an autosomal dominant genetic disease with complete penetrance, described for the first time in 1960 by Mary Holt and Samuel Oram [5]. The estimated frequency of Holt-Oram syndrome is 1/100,000 births. It is due to mutations in the *TBX3* and *TBX5* genes on chromosome 12q2 [3]. The skeletal abnormalities affect the upper limbs from phocomelia (10% of cases) to minor restriction of movement of the thumbs, elbows or shoulder [6]. The defects may be unilateral or bilateral, however radius is always affected. In majority of cases the disturbances are observed on the left side of the body [6]. The most common cardiac anomaly is secundum atrial septal defects, but also ventricular septal defects and conduction abnormalities are frequently found [4]. More severe cardiac abnormalities have been also described, including left heart hypoplasia, coarctation of the aorta, or conotruncal defects such as tetralogy of Fallot, common arterial trunk, double outlet right ventricle [7, 8].

Prenatal diagnosis can be performed even before 14th week of pregnancy and should not cause problems, especially if major limb reductions are present [9]. This condition can appeared in the first trimester with increased nuchal translucency thickness [10]. It is important that when cardiovascular and upper limb defects are detected, other syndromes such as chromosomal defects (trisomy 13, trisomy 18 and the l3q-syndrome), the TAR syndrome, Fanconi aplastic anemia (both autosomal recessive diseases) and sporadic cases of the V A TER association must be excluded [9]. In the second trimester, the use of 3D sonography allowed depiction of the upper limb anomalies very clearly [10].

In summary, Holt-Oram syndrome may be suspected when upper-extremity malformations involving radial, thenar or carpal bones, coexist with congenital heart malformations. In our case the only heart malformation detected was a right atrial enlargement. Thus, when such a finding is detected in a fetus, an accurate control of the upper limbs is necessary and a genetic test for Holt-Oram syndrome should be discussed with parents. Prenatal diagnosis of the syndrome allows to plan in advance all the necessary treatments at the time of birth.

## Supplementary Material

Supplementary Material
